# Prevalence of Back Pain in Sports: A Systematic Review of the Literature

**DOI:** 10.1007/s40279-016-0645-3

**Published:** 2016-12-29

**Authors:** Katharina Trompeter, Daniela Fett, Petra Platen

**Affiliations:** Department of Sports Medicine and Sports Nutrition, Ruhr-University Bochum, Gesundheitscampus Nord Haus 10, 44801 Bochum, Germany

## Abstract

**Background:**

Back pain is a frequent health problem in the general population. The epidemiology of back pain in the general population is well researched, but detailed data on the prevalence and risk factors of back pain in athletes are rare.

**Objective:**

The primary objective was to review articles about back pain in athletes to provide an overview of its prevalence in different sports and compare its prevalence among various types of sports and the general population.

**Data Sources:**

A comprehensive search of articles published through May 2015 was conducted. Two independent reviewers searched six databases from inception (PubMed^®^, Embase, MEDLINE^®^, Cochrane Library, PsycINFO and PSYNDEX), using specifically developed search strategies, for relevant epidemiological research on back pain in 14- to 40-year-old athletes of Olympic disciplines. The reviewers independently evaluated the methodological quality of reviewed articles meeting the inclusion criteria to identify potential sources of bias. Relevant data were extracted from each study.

**Results:**

Forty-three articles were judged to meet the inclusion criteria and were included in the assessment of methodological quality. Of these, 25 were assessed to be of high quality. Lifetime prevalence and point prevalence were the most commonly researched episodes and the lower back was the most common localization of pain. In the high-quality studies, lifetime prevalence of low back pain in athletes was 1–94%, (highest prevalence in rowing and cross-country skiing), and point prevalence of low back pain was 18–65% (lowest prevalence in basketball and highest prevalence in rowing).

**Conclusion:**

The methodological heterogeneity of the included studies showed a wide range of prevalence rates and did not enable a detailed comparison of data among different sports, within one discipline, or versus the general population. Based on the results of this review, however, it seems obvious that back pain requires further study in some sports.

**Electronic supplementary material:**

The online version of this article (doi:10.1007/s40279-016-0645-3) contains supplementary material, which is available to authorized users.

## Key Points

**Table:** 

Back pain is a frequent health problem in athletes.
The prevalence rates of back pain in athletes vary enormously.
Validated instruments and consideration of seasonality are needed in further studies to determine prevalence rates of back pain in sports.

## Introduction

Back pain, especially low back pain is a frequent health problem in the general population. It can cause disability, reduce the quality of life, and impair ability to work, which constitutes a great socioeconomic burden on patients and society [1]. It is also the leading cause of limitation of activity and absence from work throughout most parts of the world [2–6], and results in enormous costs for the healthcare system.

In the general population, the epidemiology of back pain and low back pain is well researched, but due to the methodological heterogeneity among studies, a wide range of prevalence has been reported for different groups over time. Lifetime prevalence for the general population has been reported to be as high as 85% [7, 8]. One-year prevalence of low back pain ranges from 1 to 83% and point prevalence from 1 to 58% [3, 9]. Nevertheless, an accurate estimate of prevalence is necessary to assess the impact of back pain in the population and is an important basis for etiologic studies and healthcare evaluations [3].

The relationship between low back pain and physical activity has also been well researched [10, 11]. The importance of physical activity in the treatment of low back pain is generally accepted. However, an increase in physical activity has been suggested to be both a preventive factor and a possible risk factor for low back pain. There is evidence for an association between high physical workloads and back injury. For example, occupational exposure, strenuous workloads, frequent lifting, bending and twisting, and extreme sports activities are well-recognized risk factors for low back pain [10–13]. At the same time, it is suggested that an inactive or sedentary lifestyle is associated with low back pain complaints. Studies focusing on physical activity and low back pain indicate that the relationship between activity level and low back pain follows a U-shaped curve [11, 14, 15]. Many studies have shown that both too little and too much activity is harmful to spinal health [10, 11, 16–20], but the relationship between sports and spinal health has not been adequately clarified. Elite athletes have a higher grade of physical activity and thus might have a higher risk of developing back pain. They spend much time in training and competition, which subjects their bodies to a great deal of mechanical strain and, thus, a high level of stress on the musculoskeletal system. Depending on the sports discipline, this stress is exceedingly high especially in the years from adolescence (14 years of age), in which elite competitive sports begin, until peak competitive performance at ages of up to 40 years [21]. The amount of strain on the back depends on the duration, intensity, and frequency of training, the type of sport, the level of competition, and the training periods during the year. However, the exact influence of this daily strain on back pain is not known. It is well known that sports participation generally influences health in a positive way [11], but there is a lack of knowledge about the optimal dose-effect relation. As in the general population, back pain in athletes can lead to high costs of treatment, dropping out of training and competition, decreased quality of life, and limitations to performance [22]. In this context, back pain is a relevant topic for sports medicine professionals as well as for athletes, coaches, and physiotherapists. Of particular concern is whether sports, and what types of sports, are associated with a higher or lower prevalence of back pain compared with other sports; how the training and competition level affect the prevalence of back pain; and the general population. This information would facilitate identification of possible risk factors and the development of prevention strategies in special-risk sport groups. At present, the prevalence of back pain, especially low back pain, with respect to sport-specific loads and types of sports remains unclear.

The purpose of this study was to review articles on back pain in athletes ranging from adolescence (14 years of age) to the maximal age of peak competitive performance (40 years of age) to more precisely determine the prevalence rates of back pain in different sports, compare the prevalence of back pain in different types of sports, and compare these with the general “non-sporting” population.

## Methods

Details of the search strategy method, inclusion criteria, analysis method, and data extraction form were specified beforehand and documented in a protocol. This protocol was not modified during the study to restrict the likelihood of biased post-hoc decisions, such as selective outcome reporting.

### Search Strategy

A systematic review of the literature was performed in accordance with the Preferred Reporting Items for Systematic Review and Meta Analyses (PRISMA) statement using the PRISMA checklist [23]. From 1 January 2015 to 31 May 2015, two independent researchers (KT and DF) undertook a comprehensive computerized search regarding the prevalence of back pain in sports. Athletes were defined as 14- to 40-year-old individuals participating in competitions of an Olympic discipline at any competition level. Six databases (PubMed^®^, Embase, MEDLINE^®^, Cochrane Library, PsycINFO, and PSYNDEX) were searched electronically from inception using the terms ‘back pain’, ‘neck pain,’ and ‘spine’ occurring in combination with the terms ‘sports’ AND ‘prevalence.’ Additionally, these terms were combined with terms of different Olympic sports (‘alpine skiing’ OR ‘aquatics’ OR ‘archery’ OR ‘badminton’ OR ‘basketball’ OR ‘boxing’ OR ‘biathlon’ OR ‘bobsleighing’ OR ‘canoe’ OR ‘cross-country skiing’ OR ‘curling’ OR ‘cycling’ OR ‘equestrian’ OR ‘fencing’ OR ‘figure skating’ OR ‘football’ OR ‘freestyle skiing’ OR ‘golf’ OR ‘gymnastics’ OR ‘handball’ OR ‘hockey’ OR ‘horse riding’ OR ‘ice hockey’ OR ‘judo’ OR ‘luge’ OR ‘Nordic combined’ OR ‘pentathlon’ OR ‘rugby’ OR ‘running’ OR ‘sailing’ OR ‘shooting’ OR ‘short track’ OR ‘ski jumping’ OR ‘snowboarding’ OR ‘soccer’ OR ‘speed skating’ OR ‘swimming’ OR ‘table tennis’ OR ‘taekwondo’ OR ‘tennis’ OR ‘track and field’ OR ‘trampoline’ OR ‘triathlon’ OR ‘volleyball’ OR ‘water polo’ OR ‘wrestling’ OR ‘weightlifting’). Each database automatically uses its own term mapping. The exact search strategy used in the present study is shown in Electronic Supplementary Material Table S1. The results were screened to identify relevant studies, first by title, next by abstract, and finally by full text. Non-relevant titles and abstracts were omitted. Full texts were screened regarding the inclusion criteria and were included in the review only if they met all criteria. Differences in search outcomes were verified and consensus for inclusion was reached. All English- or German-language articles investigating the occurrence of back pain in sports and published before 31 May 2015 were identified for this review, and all reference lists of selected articles were checked for other relevant articles.

### Inclusion and Exclusion Criteria

Studies investigating the occurrence of back or neck pain in sports were identified. There was no limitation on how this was measured or with regard to study design. Other inclusion criteria were:

1. Full report published in a scientific journal;
2. Study written in English or German;
3. Sample represented athletes participating in an Olympic sport;
4. Study examined sport-specific prevalence rates;
5. Age of sample between 14 and 40 years;
6. Outcome included the association between sports and the presence of cervical, thoracic, or lumbosacral pain using one of the following terms: ‘back pain’, ‘cervical pain’, ‘neck pain’, ‘thoracic pain’, ‘upper back pain’, ‘lumbar pain’, ‘lumbosacral pain’, ‘lower back pain’, or ‘low back pain’.

Letters and abstracts, studies investigating the general population and medical patients, and studies investigating pain from a specific cause (i.e., traumatic injury) were excluded.

### Assessment of Methodological Quality

To explore the heterogeneity of the study results, we hypothesized before conducting the analysis that prevalence rates of back pain may differ according to the methodological quality of the studies. Thus, we decided that a minimum requirement for meaningful data collection and interpretation had to be reached to reduce the risk of bias [2]. Selected articles that met the inclusion criteria were evaluated for methodological quality by applying a critical-appraisal tool (Table 1), which was devised by Leboeuf-Yde and Lauritsen [2]. The original tool consisted of 11 criteria. Walker [3] subsequently modified this tool by adding one additional criterion. It was used in previous reviews examining the prevalence of back pain and uses three methodological tests containing 12 criteria for prevalence studies [2–4]. The criteria are related to the representativeness of the study sample, quality of data, and definition of back pain. The criteria were verified for their presence (criterion fulfilled) or absence (criterion not fulfilled) in the studies. To assess methodological quality, each study was given a methodological score, expressed as the proportion of fulfilled criteria out of the total number of criteria. The mean methodological score of the studies was 69% [standard deviation (SD) 17%; range 30–100%] and was used to estimate a cutoff-point to gain insight into the risk of bias within the results [4]. According to other epidemiological reviews that used the same critical appraisal tool [3, 4], this point was arbitrarily set marginally lower than that mean, thus, the authors considered a score of 65% and above to indicate a high-quality study. Studies with a score <65% were considered to be of low quality. Only high-quality studies were included in this review. Two reviewers (KT and DF) independently assessed the quality of each study. Disagreement between the reviewers on individual items was discussed until consensus was reached. Overall between-reviewer agreement per item of the critical appraisal tool was calculated by unweighted kappa statistic (*κ*), with values between 0.61 and 0.80 considered substantial agreement and values between 0.81 and 1.00 considered almost perfect agreement [24].

**Table 1:** Study methodological quality critical appraisal tool

**A: Is the final sample representative of the target population?**
1. At least one of the following must apply to the study: an entire target population, randomly selected sample, or sample stated to represent the target population
2. At least one of the following: reasons for nonresponse described, nonresponders described, comparison of responders and nonresponders, or comparison of sample and target population
3. Response rate and, if applicable, drop-out rate reported
**B: Quality of the data?**
4. Were the data primary data of back pain or were they taken from a survey not specifically designed for that purpose?
5. Were the data collected from each adult directly or were they collected from a proxy?
6. Was the same mode of data collection used for all subjects?
7. At least one of the following in the case of a questionnaire: a validated questionnaire or at least tested for reproducibility
8. At least one of the following in the case of an interview: interview validated, tested for reproducibility, or adequately described and standardized
9. At least one of the following in the case of an examination: examination validated, tested for reproducibility, or adequately described and standardized
**C: Definition of back pain**
10. Was there a precise anatomic delineation of the back area or reference to an easily obtainable article that contains such specification?
11. Was there further useful specification of the definition of back pain, or question(s) put to study subjects quoted such as the frequency, duration, or intensity, and character of the pain. Or was there reference to an easily obtainable article that contains such specification?
12. Were recall periods clearly stated: e.g., 1 week, 1 month, or lifetime?

### Data Extraction and Analysis

A data extraction sheet was pilot-tested independently from both reviewers on ten included studies and refined accordingly. In the preparation of the systematic review, one reviewer independently extracted defined data from the included studies, and the other checked the data of each study. Disagreements were resolved by discussion between the two reviewers. If no agreement could be reached, it was planned that a third author would decide. The first author, country, explored sports discipline, final sample size, age and sex distributions, sports level, response rate, collection mode, definition of pain, pain localization, recall periods, prevalence data, and calculated risk factors were extracted from each study. This was separately conducted for the high- and low-quality studies. The original authors were not contacted for further details.

All extracted data were rounded to the nearest integer. The analysis of prevalence data refers only to high-quality studies. Additionally, the studies were evaluated with respect to the confounders’ age and sex.

### Data Pooling

Studies that used exactly the same instrument for data collection were summarized and the results were pooled if studies reported the same time periods and the same localization of pain. The overall prevalence rate of back pain for all athletes within these pooled data was calculated considering the sample sizes. To achieve this, the total number of athletes reporting back pain was calculated for each study. This was done using information on sample size and prevalence rate in percentages. The total number of athletes as well as the total number of athletes with back pain were then calculated. The overall prevalence rate was calculated using this information.

## Results

### Search Strategy

**Fig. 1:**
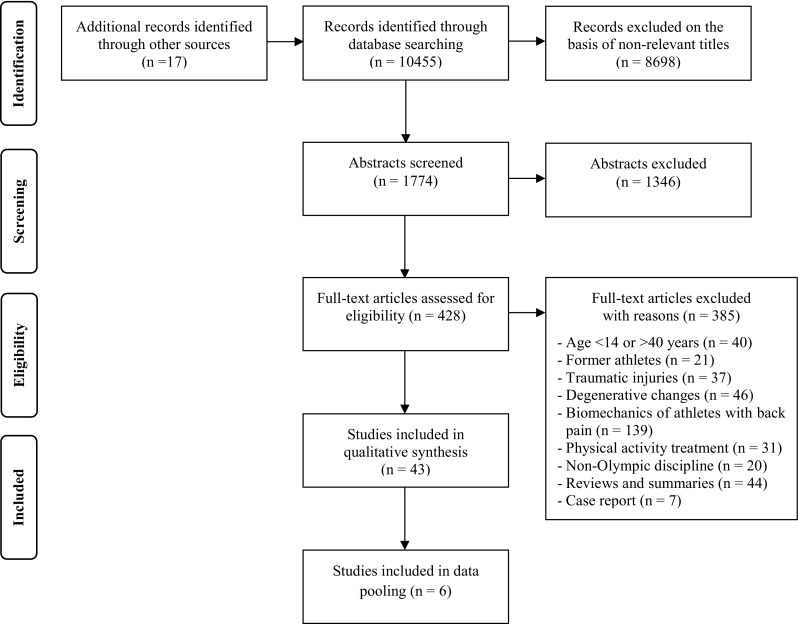
PRISMA flow diagram

The comprehensive computerized search for published epidemiological research with regard to prevalence of back pain in sports (Fig. 1) achieved 10,455 hits. In addition, 17 relevant articles were identified by checking the reference lists of selected articles. First, all titles were screened and 8698 non-relevant titles were omitted. In the next step 1774 abstracts were screened according to their relevance. Of these, 1346 were excluded, mainly because of failure to examine an athletic population. For example, many studies examined physical activity treatments in back pain patients after rehabilitation. After exclusion of these 1346 abstracts, 428 studies were considered eligible for full-text screening. Full-text screening led to the exclusion of 385 studies on the basis of disagreement with the inclusion criteria. Reasons for exclusion are shown in Fig. 1. Ultimately, 43 studies were included in the qualitative synthesis.

### Methodological Assessment

Forty-three articles were judged to have met the inclusion criteria and were included in the methodological-quality assessment (Table 2). Agreement per item between the two independent reviewers was 92% (472/516 items), which resulted in almost perfect agreement (*κ* = 0.855; standard error: 0.021).

**Table 2:** Quality assessment of trials meeting the inclusion criteria

	Representative			Quality of data						Definition of back pain			Total score (%)
	1	2	3	4	5	6	7	8	9	10	11	12	
Aggrawal et al. [25]	CNF	CNF	CNF	CF	CF	CF	NA	NA	CNF	CNF	CF	CNF	40
Alricsson and Werner [50]	CF	CNF	CF	CF	CF	CF	CF	NA	NA	CF	CF	CF	90
Bahr [32]	CNF	CNF	CF	CF	CF	CF	CF	NA	NA	CF	CF	CF	80
Bahr et al. [29]	CF	CF	CF	CF	CF	CF	CF	NA	NA	CF	CF	CF	100
Baranto et al. [49]	CF	CF	CF	CF	CF	CF	CF	NA	NA	CF	CF	CF	100
Bergstrøm et al. [71]	CF	CNF	CF	CF	CF	CF	NA	NA	CF	CNF	CF	CNF	70
Brynhildsen et al. [34]	CF	CNF	CF	CF	CF	CF	CNF	NA	NA	CF	CF	CF	80
Brynhildsen et al. [72]	CF	CNF	CNF	CF	CF	CF	CNF	NA	NA	CF	CNF	CF	60
Cali et al. [52]	CF	CF	CNF	CF	CF	CF	NA	NA	CF	CF	CF	CF	90
Cabri et al. [30]	CNF	CNF	CF	CF	CF	CF	CF	NA	NA	CF	CF	CF	80
Clarsen et al. [44]	CNF	CNF	CF	CF	CF	CF	NA	CF	NA	CF	CF	CF	80
Dubravcic-Simunjak et al. [55]	CF	CNF	CF	CNF	CF	CF	CNF	NA	NA	CF	CF	CF	70
Eriksson et al. [43]	CF	CNF	CF	CF	CF	CF	CNF	NA	NA	CF	CF	CF	80
Greene et al. [73]	CNF	CNF	CNF	CF	CF	CF	CNF	NA	NA	CF	CF	CF	60
Hangai et al. [40]	CNF	CNF	CNF	CF	CF	CF	CNF	NA	NA	CF	CF	CF	60
Haydt et al. [47]	CNF	CNF	CNF	CF	CF	CF	CF	NA	NA	CF	CF	CF	70
Howell [57]	CNF	CNF	CNF	CF	CF	CF	CNF	NA	NA	CF	CF	CF	60
Hutchinson [33]	CNF	CNF	CNF	CF	CF	CF	NA	NA	NA	CF	CF	CF	67
Iwamoto et al. [39]	CF	CNF	NA	CF	CF	CF	NA	NA	CNF	CF	CF	CF	78
Kaneoka et al. [38]	CNF	CNF	CNF	CF	CF	CF	CNF	NA	NA	CF	CF	CF	60
Kernahan et al. [74]	CNF	CNF	CNF	CF	CF	CF	CNF	NA	NA	CNF	CF	CF	50
Koyama et al. [36]	CNF	CNF	CNF	CF	CF	CF	CF	NA	NA	CF	CNF	CF	60
Lindgren and Twomey [75]	CNF	CNF	CNF	CF	CF	CF	CNF	NA	NA	CF	CF	CF	60
Lively [42]	CNF	CNF	NA	CF	CF	CF	NA	NA	CF	CF	CF	CF	78
Maselli et al. [28]	CF	CNF	CF	CF	CF	CF	CF	NA	NA	CF	CF	CF	90
Martins et al. [41]	CNF	CNF	CNF	CNF	CF	CF	CNF	NA	NA	CNF	CNF	CF	30
Mulhearn and Georg [76]	CNF	CNF	CNF	CF	CF	CF	CNF	NA	NA	CF	CF	CF	60
Murtaugh [35]	CNF	CNF	CF	CF	CF	CF	CNF	NA	NA	CNF	CNF	CF	50
Newlands et al. [27]	CF	CNF	CF	CF	CF	CF	CNF	NA	NA	CF	CF	CF	80
Ng et al. [45]	CF	CNF	CF	CF	CF	CF	CNF	NA	NA	CF	CF	CF	80
Okada et al. [37]	CNF	CNF	CNF	CF	CF	CF	CF	NA	NA	CF	CF	CNF	60
Perich et al. [53]	CNF	CNF	CF	CF	CF	CF	CF	NA	NA	CF	CF	CF	80
Reilly and Seaton [77]	CNF	CNF	CF	CF	CF	CF	CNF	NA	NA	CNF	CNF	CF	50
Roy et al. [51]	CNF	CNF	CNF	CF	CF	CF	CF	NA	NA	CF	CF	CF	70
Selanne et al. [54]	CNF	CNF	CF	CF	CF	CF	CF	NA	NA	CF	CF	CF	80
Swärd et al. [46]	CF	CNF	CNF	CF	CF	CF	CNF	NA	NA	CF	CF	CF	70
Swärd et al. [48]	CF	CF	CNF	CF	CF	CF	NA	CF	NA	CF	CF	CF	90
Szot et al. [78]	CNF	CNF	CNF	CF	CF	CF	NA	NA	CNF	CNF	CNF	CNF	30
Tunas et al. [31]	CNF	CF	CF	CF	CF	CF	CF	NA	NA	CF	CF	CF	90
van Hilst et al. [26]	CNF	CNF	CF	CF	CF	CF	CF	NA	NA	CF	CF	CF	90
Vad et al. [79]	CNF	CNF	NA	CF	CF	CF	NA	NA	CNF	CF	CF	CNF	56
Vad et al. [80]	CNF	CNF	CNF	CF	CF	CF	NA	CNF	NA	CF	CF	CF	60
Willscheid et al. [81]	CNF	CNF	CF	CNF	CF	CF	CNF	NA	NA	CNF	CNF	CF	40

*CF* criterion fulfilled, *CNF* criterion not fulfilled, *NA* not applicable

See Table 1 for definitions of 1 to 12

Twenty-five articles had a score of 65% or more and were thus assessed as high-quality studies. The most common methodological deficits were related to representativeness of the sample (65%), information about nonresponders (88%), response and drop-out rates (50%), and no valid or adequately described and standardized method in case of a questionnaire (56%) or examination (57%). In 19% of the studies there was no mention of any anatomic delineation of the back area.

### General Description of High- and Low-Quality Studies

Descriptive data extracted from the 25 high-quality studies are represented as an overview summary in Tables 3 and 4. The 18 low-quality studies can be seen in Electronic Supplementary Material Table S2. All high- and low-quality studies were published between 1979 [25] and 2015 [26–28] and employed various modes of data collection, including questionnaires, interviews, examinations, and medical reports. Questionnaires were the most common method for data collection (32 studies); 14 of the 32 used validated questionnaires and six used the Nordic Questionnaire [26, 28–32]. The final sample size of Olympic disciplines ranged from seven [33] to 361 [34]. Nineteen studies reported a response rate varying between 32% [28] and 100% [26, 29, 35], mean response rate was 81%. Recall periods varied from present to lifetime and described a full array of prevalence data, and most (22 studies) reported lifetime prevalence. Point prevalence was defined as pain at the time of examination or during the last 7 days.

Five studies were from Japan [36–40], one was from Brazil [41], and one was from India [25], the remainder were from Western countries.

Prevalence data for 26 different Olympic disciplines were extracted. The most frequently investigated disciplines were soccer, gymnastics, rowing, and field hockey, with nine, eight, seven, and six publications, respectively.

**Table 3:** Characteristics of high-quality studies included in the review (Summer Olympic disciplines)

References	Country	Final sample size	Age [years] mean ± SD^a^ (range)	Level	Response rate [%]	Collection mode	Definition of pain	Localization	Recall periods	Prevalence [%]
**Basketball**										
Brynhildsen et al. [34]	Sweden	150 (F)	Med: 21 (16–31)	Swedish first league clubs	85	Q	Subjective feeling of back pain; if she had suffered from low back pain ever (previous) or during the last wk (current)	LB	LT, 7-d	LT: 53, 7-d: 21
Cabri et al. [30]	Germany	100	20.4 (15–35)	Regional/national squat/selection	50	Q	Pain, ache, or discomfort in the lower back with or without radiation to one or both legs	B, LB, TH, C	LT, 1-yr, 7-d	LT: B: 76, LB: 46, TH: 17, C: 22; 1-yr: LB: 35, TH: 20, C: 21; 7-d: LB: 18, TH: 6, C: 4
**Cycling**										
Clarsen et al. [44]	Norway	109	26 ± 4	UCI World Tour/Tour de France level, UCI Europe Tour level	94	In	Pain, ache, or soreness in the low-back with or without radiating pain to the gluteal area or lower extremities	LB	LT, 1-yr	LT: 65, 1-yr: 58
**Dancing**										
Lively [42]	USA	31	19 (17–22)	Student-athletes	–	E	Pre-existing recurrent low back pain was defined as lumbar pain on at least two previous occasions that required the athlete to suspend participation in sports for at least one day	LB	LT	3
**Field hockey**										
Haydt et al. [47]	USA	90 (F)	19 ± 1 (18–22)	NCAA Division III intercollegiate	–	Q	low back pain lasting more than 24 h, not associated with menstruation	LB	I	56
van Hilst et al. [26]	The Netherlands	T: 61		Elite, highest level in their age category	81	Q	Pain, ache, or discomfort in the region of the lower back whether or not it extended from there to one or both legs (sciatica), lower back region was indicated as a shaded area in a drawn picture of the upper body	LB	12-mo	56 33 67
		21 (M)	17 (15–24)							
		40 (F)	16 (14–19)							
**Gymnastics**										
Lively [42]	USA	21	19 (17–22)	Student-athletes	–	E	Pre-existing recurrent low back pain was defined as lumbar pain on at least two previous occasions that required the athlete to suspend participation in sports for at least one day	LB	LT	10
Swärd et al. [48]	Sweden	24 (M)	Med: 23 (19–29)	Present or previous members of the Swedish national team	–	In	Previous or present pain in the thoracic or lumbar part of the back with a duration of 1 wk or more or recurrent pain irrespective of its duration	B	LT, PP	LT: 79, PP: 38
Swärd et al. [46]	Sweden	26 (M)	Med: 19 (16–25)	Current or recent members of the national team or the national junior team	–	Q	Previous or present pain located in the thoracic or lumbar spine with a duration of more than 1 wk, or recurrent pain irrespective of duration	B	LT I	85
		26 (F)	Med: 16 (14–25)							65
**Handball**										
Tunas et al. [31]	Norway	190 (F)	22 ± 3 (18–32)	Elite	99	Q	Pain, ache or discomfort in the lower back with or without radiation to one or both legs	LB	LT, 12-mo, 7-d	LT: 63, 12-mo: 59, 7-d: 26
**Orienteering**										
Bahr et al. [29]	Norway	227 (M: 129, F: 98)	M: 24 ± 7 F: 23 ± 6	Qualified for national championships	99	Q	Pain, ache, or discomfort in the low back with or without radiation to one or both legs (sciatica)	LB	LT, 12-mo, 7-d	LT: 57, 12-mo: 50, 7-d: 19
Baranto et al. [49]	Sweden	18	Med: 25 (20–35)	Swedish top male athletes, active at least since the age of 10 years	–	Q	Previous or present pain located in the thoraco-lumbar spine	B	LT	56
**Rhythmic gymnastics**										
Hutchinson [33]	USA	7	16 (15–17)	US national team members		DMR	–	B	7-wk	86
**Rowing**										
Bahr et al. [29]	Norway	199 (M: 131, F: 68)	M: 21 ± 6 F: 22 ± 5	Qualified for national championships	100	Q	Pain, ache, or discomfort in the low back with or without radiation to one or both legs (sciatica)	LB	LT, 12-mo, 7-d	LT: 63, 12-mo: 55, 7-d: 25
Maselli et al. [28]	Italy	133 (M: 107, F: 26)	Med: 19 (16–33)	National elite level, all athlete qualified for national championship, TV: 14 (6–20) h/wk	32	Q	Pain, ache, or discomfort in the low back with or without radiation to one or both legs (sciatica)	LB	LT, 1-yr, 1-mo	LT: 65, 1-yr: 41, 1-mo: 20
Newlands et al. [27]	New Zealand	76 (M: 46; F: 30)	M: 23 ± 4 F: 21 ± 4	Elite, represented New Zealand at a World Championship event in 2011, years of rowing: 8 ± 4 (M), 6 ± 3 (F)	75	Q	Pain, ache or discomfort in the low back with or without referral to the buttocks or legs that has been present for more than 1 wk and/or interrupted at least one training session	LB	1-yr, 1-mo (monthly in a 1-year period)	1-yr: 53, 1-mo: 6-25
Ng et al. [45]	Australia	130 (M) 235 (F)	14–16	Rowers who competed for independent boys and girls schools	42 72	Q	Pain located between L1 and gluteal folds and this area of the body was shown in a visual manner	LB	LT, PP	LT: 94, PP: 65 LT: 80, PP: 53
Perich et al. [53]	Australia	221 (F), 90 intervention, 131 control group	14–17	TV: 7 h/wk school-based rowing programme	–	Q	–	LB	PP	37 intervention group 32 rowing control group
Roy et al. [51]	USA	23 (M)	20.3 ± 1.3	Varsity rowers	–	Q	A single or recurring incidence of low back pain during the past year which interfered with activities of daily living, including rowing or training activities	LB	1-yr	26
**Rugby**										
Iwamoto et al. [39]	Japan	327	(15–16)	High school	–	E	Non-traumatic low back pain that resulted in stopping playing rugby completely for at least 1 day	LB	LT I	29
**Soccer**										
Brynhildsen et al. [34]	Sweden	361 (F)	Med: 21 (14–36)	Swedish first league clubs	85	Q	Subjective feeling of back pain; if she had suffered from low back pain ever (previous) or during the last wk (current)	LB	LT, 7-d	LT: 42, 7-d: 32
Çali et al. [52]	Turkey	T: 121 (M)	23.8 ± 4.1 (16–34)	Professional players, Turkish Super league, TV: 6 d/wk, 90 min/training unit	–	E	–	LB	12-mo	31
		12 goalkeeper								33
		34 defender								26
		52 midfielder								37
		23 forward								24
Lively [42]	USA	153	19 (17–22)	Student-athletes	–	E	Pre-existing recurrent low back pain was defined as lumbar pain on at least two previous occasions that required the athlete to suspend participation in sports for at least one day	LB	LT	1
Swärd et al. [46]	Sweden	31 (M)	Med: 21 (18–25)	Members of one of the two top-ranked first division soccer teams, the majority were members of the national team or the national under-21 team	–	Q	Previous or present pain located in the thoracic or lumbar spine with a duration of more than 1 wk, or recurrent pain irrespective of duration	B	LT I	58
Tunas et al. [31]	Norway	277 (F)	22 ± 4 (18–32)	Elite	98	Q	Pain, ache or discomfort in the lower back with or without radiation to one or both legs	LB	LT, 12-mo, 7-d	LT: 61, 12-mo: 57, 7-d: 24
van Hilst et al. [26]	The Netherlands	45 (M)	18 (16–19)	Elite, highest level in their age category	100	Q	Pain, ache, or discomfort in the region of the lower back whether or not it extended from there to one or both legs (sciatica), lower back region was indicated as a shaded area in a drawn picture of the upper body	LB	12-mo	64
**Swimming**										
Lively [42]	USA	84	19 (17–22)	Student-athletes	–	E	Pre-existing recurrent low back pain was defined as lumbar pain on at least two previous occasions that required the athlete to suspend participation in sports for at least one day	LB	LT	2
**Tennis**										
Lively [42]	USA	35	19 (17–22)	Student-athletes	–	E	Pre-existing recurrent low back pain was defined as lumbar pain on at least two previous occasions that required the athlete to suspend participation in sports for at least one day	LB	LT	3
Swärd et al. [46]	Sweden	30 (M)	Med: 20 (17–25)	20: ranked in the top 30 in Sweden, including members of the Davis Cup team 1984-85, and 10: ranked between 30 and 70 in Sweden	–	Q	Previous or present pain located in the thoracic or lumbar spine with a duration of more than 1 wk, or recurrent pain irrespective of duration	B	LT I	50
**Track and field**										
Lively [42]	USA	116	19 (17–22)	Student-athletes	–	E	Pre-existing recurrent low back pain was defined as lumbar pain on at least two previous occasions that required the athlete to suspend participation in sports for at least one day	LB	LT	2
**Volleyball**										
Bahr [32]	Norway	57 (M)		Qualified for main draw at FIVB World Tour Grand Slam tournament 2008	90	Q	Pain, ache or soreness in the low back, with or without radiating pain in the gluteal area or the lower extremity	LB	2-mo, 7-d	2-mo: 46, 7-d: 32
		58 (F)								2-mo: 40, 7-d: 22
Brynhildsen et al. [34]	Sweden	205 (F)	Med: 22 (16–35)	Swedish first league clubs	85	Q	Subjective feeling of back pain; if she had suffered from low back pain ever (previous) or during the last wk (current)	LB	LT, 7-d	LT: 63, 7-d: 34
Lively [42]	USA	24	19 (17–22)	Student-athletes	–	E	Pre-existing recurrent low back pain was defined as lumbar pain on at least two previous occasions that required the athlete to suspend participation in sports for at least one day	LB	LT	8
**Weight lifting**										
Baranto et al. [49]	Sweden	21	Med: 30 (18–40)	Swedish top male athletes, active at least since the age of 10 years	–	Q	Previous or present pain located in the thoraco-lumbar spine	B	LT	71
**Wrestling**										
Baranto et al. [49]	Sweden	13	Med: 24 (22–41)	Swedish top male athletes, active at least since the age of 10 years	–	Q	Previous or present pain located in the thoraco-lumbar spine	B	LT	77
Lively [42]	USA	61	19 (17–22)	Student-athletes	–	E	Pre-existing recurrent low back pain was defined as lumbar pain on at least two previous occasions that required the athlete to suspend participation in sports for at least one day	LB	LT	3
Swärd et al. [46]	Sweden	29 (M)	Med: 20 (17–25)	National (or junior) team or were active in the First Division of the National Wrestling League	–	Q	Previous or present pain located in the thoracic or lumbar spine with a duration of more than 1 wk, or recurrent pain irrespective of duration	B	LT I	69

*B* back, *C* cervical spine, *NCAA* National Collegiate Athletic Association, *d* day, *DMR* daily medical report, *E* examination, *F* female, *FIVB* Fédération Internationale de Volleyball, *h* hour, *In* interview, *I* Incidence, *LB* low back, *L1* first lumbar vertebra, *LT* lifetime, *M* male, *min* minutes, *mo* month, *Med* median, *N* neck, *PP* point prevalence, *Q* questionnaire, *SD* standard deviation, *T* total, *TH* thoracic spine, *TV* training volume, *UB* upper back, *UCI* Union Cycliste Internationale, *USA* United States of America, *US* United States, *wk* week, *yr* year

^a^Except where otherwise indicated

**Table 4:** Characteristics of high-quality studies included in the review (Winter Olympic disciplines)

References	Country	Final sample size	Age [years] mean ± SD^a^ (range)	Level	Response rate [%]	Collection mode	Definition of pain	Localization	Recall periods	Prevalence [%]
**Cross-country skiing**										
Alricsson and Werner [50]	Sweden	120 (M: 58%, F: 42%)	18 ± 1	Top national level	92	Q	Previous or present pain while skiing	B, LB, TH, C	LT, 3-mo	LT: B: 47, LB: 44, TH: 7, C: 3; 3-mo: B: 26, C: 11
Bahr et al. [29]	Norway	257 (M: 165, F: 92)	M: 23 ± 5 F: 21 ± 4	Qualified for national championships	100	Q	Pain, ache, or discomfort in the low back with or without radiation to one or both legs (sciatica)	LB	LT, 12-mo, 7-d	LT: 65, 12-mo: 63, 7-d: 24
Eriksson et al. [43]	Sweden	T: 87	21 (16–26)	Ski high school, top national level in their age groups	91	Q	Previous or present recurrent skiing correlated with backache that more or less affected skiing ability	B, LB, TH, C	LT while skiing	B: 64, LB: 61, TH: 6, C: 8
		53 (M)								B: 68, LB: 66, TH: 6, C: 6
		34 (F)								B: 59, LB: 53, TH: 6, C: 12
**Figure skating**										
Dubravic-Simunjak et al. [55]	Croatia	T: 469	13–20 Med: 18 (M) Med: 16 (F)	World Figure Skating Championships	82	Q	–	LB	During junior skating career	9
		Singles: 104 (M)								15
		Singles: 107 (F)								13
		Pairs: 61 (M)								12
		Pairs: 61 (F)								8
		Ice dancing: 68 (M)								0
		Ice dancing: 68 (F)								0
**Ice hockey**										
Baranto et al. [49]	Sweden	19	Med: 24 (19–31)	Swedish top male athletes, active at least since the age of 10 years	–	Q	Previous or present pain located in the thoraco-lumbar spine	B	LT	90
Selanne et al. [54]	Finland	121 (M)	15 (14–16)	National level	93	Q	Pain occurred at least once a month during the previous 3 months	LB, UB, N	3-mo	LB: 54, UB: 31, N: 44
**Skiing (alpine, cross-country)**										
Bergstrøm et al. [71]	Norway	45 (M: 21, F: 24)	17 (15–19)	High school athletes, TV: (6–20) h/wk	76	E	Back pain was reported when present in training or after training	LB, N	–	LB: 67, N: 22
**Speed skating**										
van Hilst et al. [26]	The Netherlands	T: 75		Elite, highest level in their age category	65	Q	Pain, ache, or discomfort in the region of the lower back whether or not it extended from there to one or both legs (sciatica), lower back region was indicated as a shaded area in a drawn picture of the upper body	LB	12-mo	60 54 66
		37 (M)	18 (15–23)							
		38 (F)	18 (14–25)							

*B* back, *C* cervical spine, *d* day, *E* examination, *F* female, *h* hour, *LB* low back, *LT* lifetime, *M* male, *Med* median, *mo* month, *N* neck, *Q* questionnaire, *SD* standard deviation, *T* total, *TH* thoracic spine, *TV* training volume, *UB* upper back, *wk* week

^a^Except where otherwise indicated

### Definition of Back Pain

For all high- and low-quality studies, there was no consensus regarding the definition of back pain, low back pain, thoracic pain, and neck/cervical pain. The pain differed with respect to localization, intensity, frequency, and duration. In some cases an athlete was defined as having pain only if its consequence, such as missing three days of training, was noted. Pain was defined in 29 of the 43 studies. Studies that used the term ‘back pain' either failed to identify which part of the back was involved or used it as a synonym for ‘low back pain' or meant the thoracolumbar spine.

### Analysis of Back Pain Episodes in High-Quality Studies

#### Lifetime Prevalence

Fifteen studies [28–31, 34, 39, 42–50] investigated lifetime prevalence data of athletes from 16 different Olympic sports disciplines. Six studies [30, 43, 46, 48–50] provided lifetime prevalence data for the total back. In this anatomic region, lifetime prevalence ranged from 47 to 90%. The most frequently occurring localization was to the lower back. Twelve studies [28–31, 34, 39, 42–45, 47, 50] reported the lifetime prevalence of low back pain to range from 1 to 94%. Three studies [30, 43, 50] reported a lifetime prevalence of thoracic-spine or upper-back pain ranging from 6 to 17%. The same three studies also reported a lifetime prevalence of pain in the cervical spine, ranging from 3 to 22%.

#### One-Year Prevalence

One-year prevalence of back pain was investigated in nine studies [26–31, 44, 51, 52], which all reported a prevalence of low back pain ranging from 24 to 66%. One study [30] reported rates of thoracic-spine pain and neck pain of 20 and 21%, respectively.

#### Point Prevalence

Eight studies [29–32, 34, 45, 48, 53] referred to the point prevalence of pain, usually defined as pain at the time of examination or during the last 7 days, in different areas of the back. The lower back was the most commonly occurring area of point prevalence. All eight studies collected point-prevalence data for the lower back ranging from 18 to 65%. Present pain in the thoracic spine (6%) and cervical spine (4%) was reported by one study [30].

#### Other Recall Periods

Recall periods for back pain other than lifetime, 1-year, and point prevalence were reported in 7 studies [27, 28, 32, 33, 50, 54, 55]. Newlands et al. [27] provided monthly low back pain-prevalences between 6 and 25% over a 1-year period. Maselli et al. [28] reported a 1-month prevalence of 20% for low back pain in rowers. Other authors reported 7-week (86% [33]) and 2-month (46% for males, 40% for females [32]) prevalences of back pain. Two studies provided a 3-month prevalence: Alricsson and Werner [50] reported a prevalence of 26% for back pain and Selanne et al. [54] a prevalence of 54% for low back pain.

#### Data Pooling

In six high-quality studies, information about prevalence was collected with the Nordic questionnaire (or an adaptation) for the analysis of musculoskeletal symptoms [26, 28–32]. This instrument has been validated and standardized and is therefore considered an international standard [56]. It was possible to pool the data in these studies because the same mode of data collection was used in all six.

These studies included a total of 1679 athletes (692 males, 887 females, 100 not reported) from the following disciplines: rowing (*n* = 332), soccer (*n* = 322), cross-country skiing (*n* = 257), orienteering (*n* = 227), handball (*n* = 190), volleyball (*n* = 115), basketball (*n* = 100), speed skating (*n* = 75), and field hockey (*n* = 61). All of these studies reported low back pain in different time periods. For lifetime prevalence of low back pain, the data of four studies [28–31] were pooled (range of studies: 46–65%). Data pooling was conducted for a total of 1383 athletes (532 males, 751 females). For these athletes, an overall lifetime prevalence of 61% was calculated. Five studies [26, 28–31] reported the 12-month prevalence of low back pain (range of studies: 35–63%). The pooling was conducted with data from 1564 athletes (635 males, 829 females, 100 not reported), and an overall 12-month prevalence of 55% was calculated. For point prevalence the data from four studies [29–32] were pooled (range of studies: 18–26%). Data from 1365 athletes (482 males, 783 females) were pooled and a point prevalence of 24% was calculated.

### Analysis of Age and Sex

Nine [26–28, 32, 43, 45, 46, 50, 55] of the 25 high-quality studies differentiated prevalence rates of low back pain between males and females. In six of these studies, male athletes had a higher prevalence of low back pain or a higher probability of being affected by low back pain than did female athletes. Swärd et al. [46] found significant differences between the two sexes. They reported a lifetime prevalence of 85% for male gymnasts (median age: 19 years) and 65% for female gymnasts (median age 16 years). Bahr et al. [32] calculated a 2-month prevalence of 46 and 40% for male and female volleyball players, respectively, and a 7-day prevalence of 32 versus 22%. Ng et al. [45] evaluated rowers and reported significant sex-related differences in lifetime prevalence (94% for males and 80% for females) and point prevalence (65% for males and 53% for females). Additionally, Dubravic-Simunjak et al. [55] studied figure skaters and found a higher prevalence in males (median age: 18 years) than in females (median age 16 years) (singles: males 15%, females 13%, pairs: males 12%, females 8%). Erikson et al. [43] investigated the prevalence of back pain in male and female cross-country skiers, and found a higher prevalence in males. However, this difference was not statistically significant. Maselli et al. [28] found that male rowers had a higher probability of being affected by low back pain than did female rowers [OR = 2.62; *p* = 0.03; 95% confidence interval (CI) 1.09–6.27]. However, this finding was not reflected in a study by Newlands et al. [27] (OR = 1.07; *p* = 0.81; 95% CI 0.62–1.86). Also Alricsson and Werner [50] showed no sex-related differences in prevalence of back pain in their study. Finally, van Hilst et al. [26] reported a significantly higher 12-month prevalence of low back pain in female speed skaters and field hockey players (speed skating: male 54%, female 66%, both with a mean age of 18 years; field hockey: 33 vs. 67%, with a mean age of 17 and 16 years, respectively).

For various reasons, the data of our review did not enable calculation of age-related effects over all studies. The data collection of the included studies varied widely, and the necessary data pooling was therefore not possible. In the six pooled studies, the average age of athletes varied from 19 to 24 years and the lifetime prevalence ranged from 46 to 65%. These small ranges in age and prevalence did not enable calculation of age-related effects. Additionally, it was difficult to determine a cutoff point with which to compare younger versus older athletes. Indeed, there was a wide range of ages among the included studies (14–40 years), and the mean age of athletes in most of the studies was about 18–21 years. Thus, the distribution was not large enough to show age-related effects.

## Discussion

This review systematically evaluated and analyzed the methodological quality of the existing literature on the prevalence of back pain in athletes. To the best of our knowledge, this is the first systematic review of prevalence data for back pain in an athletic population and shows that back pain is a well-known problem in Western countries. The earliest publication date in this review was 1979, indicating that back pain is not a recent problem. However, interest in studying its prevalence is increasing. Twelve studies (28%) were published in the first half of the study period (1979–1997) and 31 studies (72%) in the second half (1998–2015).

### Methodological Aspects

This review shows that measurement, definitions, and methodological quality varied greatly among the 43 reviewed high- and low-quality studies. It was often difficult to uncover facts because of unclear reporting. There is an obvious need for clear guidelines on conducting and reporting prevalence studies and to find reliable and valid instruments to measure back pain prevalence. This problem has been mentioned in other prevalence reviews [2, 3]. In the present review, only 44% of high- and low-quality studies used validated measurements, or at least measurements tested for test-retest reliability.

Attention must be also paid to the definition of back pain. Different definitions lead to different estimates of prevalence, and no definition has been generally accepted in back pain research [11]. In the present review, prevalence data (within the same time period) varied enormously because of different definitions. For instance, low prevalence would be expected for pain defined as “pain, ache, or discomfort in the low back, with or without referral to the buttocks or legs, that has been present for more than 1 week and/or interrupted at least one training session” [27]. In contrast, higher prevalence would be expected for pain defined as “pain, ache, or discomfort in the low back with or without radiation to one or both legs (sciatica)” [29], without any further considerations such as duration of pain or interruption of training. The large variation also becomes obvious when comparing discipline-specific prevalence data. In rowing, for example, depending on the definition, lifetime prevalence of low back pain varies from 63 to 94% and point prevalence ranges from 25 to 65%. Descriptions of the exact area of pain and the frequency, duration, and intensity or severity of attacks should be standardized [3, 6], which would provide opportunities for statistical summary and data pooling.

Some items scored conspicuously poorly in the assessment of study quality. For example, the representativeness of the sample scored poorly in most studies and the sample sizes and response rates varied greatly. This might have influenced the results of our investigation. Notably, when evaluating the prevalence data of such a specific group as athletes within a particular sports discipline, it can be difficult to find a group of representatives according to the tool’s definition. In some disciplines, and especially at higher competition levels, it is difficult to gain access to a representative number of athletes. Particularly during preparation for important competitions such as national or international championships, or even the Olympic Games, coaches and athletes must concentrate on optimizing the athletes’ performance and cannot tolerate any distractions.

### Prevalence Data

One of our main focuses in the present review was to compare back pain in athletes with that in the general population. Therefore, we compared our data with those of population-based reviews that summarized studies on back pain prevalence rates and, like our review, used different methods of data collection. Although a comparison of the results between population-based studies and the present study must be interpreted carefully, it might provide the first clinically useful information on the problem of back pain in athletes.

Walker [3] found that the lifetime prevalence of low back pain in the general population ranged from 11 to 84%. In our review, the lifetime prevalence of low back pain ranged from 1 to 94%. In another population-based review, Hoy et al. [5] found lifetime prevalence rates for low back pain of up to 84% with an average prevalence of 39%. In contrast, only two of 12 articles in our review showed low back pain prevalence to be low than this mean [39, 42], while the other ten articles showed a higher low back pain prevalence. Additionally, our pooled data showed a 61% lifetime prevalence rate for low back pain, which is much higher than this mean.

With respect to the low back pain point prevalence in the general population, Walker [3] found prevalence rates ranging from 7 to 33%. Population-based data provided by Hoy et al. [5] were similar, with a mean of 18%. In contrast, our review showed a minimum of 18% [30] and a maximum of 65% [45]. All of our identified studies reported point prevalence rates greater than the mean of 18% that Hoy et al. [5] calculated for the general population. Our pooled data showed a low back pain point prevalence of 24%, which is again higher than this mean. Both population-based and athlete-based prevalence studies have shown wide ranges in the results. Nevertheless, the values seem to be higher in athletes. Even if these trends do not provide sufficient basis for definite conclusions about the problem of back pain in athletes, they indicate for the first time that back pain might be a larger problem in examined disciplines than in the general population.

To enable a comparison between different populations, such as the general population and athletes of different disciplines, standardized data collection is needed. In this review, eight studies of high quality compared prevalence data of a sample with that of a control group [29, 31, 34, 47–50, 54]. These data are shown in Electronic Supplementary Material Table S3. Only four of these studies [29, 31, 48, 49] examined a randomly selected, less active control group; most of the remaining studies used age-matched control groups. Bahr et al. [29] reported significantly higher lifetime prevalence rates in skiers and rowers than in a control group. Moreover, the 12-month prevalence rate was higher for skiers than for controls. Significant differences were also found by Swärd et al. [48], Brynhildsen et al. [34], and Selanne et al. [54]. Baranto et al. [49] found an obvious difference between athletes and controls, with lifetime prevalences of 78 and 38%, respectively. They did not mention whether these results were significant. However, Tunas et al. [31] and Haydt et al. [47] did not find a significant difference in low back pain between athletes and a control group. Conversely, Alricsson and Werner [50] found back pain and neck pain to be significantly higher in a control group than in a group of cross-country skiers at the top national level. To enable better comparisons with the general population, future studies should examine representative control groups.

### Sport-Specific Prevalence of Back Pain

According to our inclusion and exclusion criteria, we found sports disciplines that received more attention regarding the prevalence of back pain (e.g., soccer, gymnastics, rowing, and field hockey). Some other sports disciplines, such as boxing, badminton, sailing, taekwondo, and table tennis, had no identified data regarding the prevalence of back pain. Because of the above-mentioned methodological problems, a comparison of the results of different studies in one sports discipline is not sensible. Thus, we cannot make a general statement regarding which types of sports are associated with a higher or lower prevalence of back pain based on this review. Furthermore, it is possible that the relevance of the issue is greater in these highly investigated sports disciplines, than in under-investigated or uninvestigated sports disciplines. This also influences the back pain prevalence data of athletes in general. When comparing values that are found for other populations, such as the general population, the possibility must be considered that mainly disciplines with a back pain problem are examined. The consideration of disciplines with a potential preventive influence on the development of back pain would accordingly lead to lower prevalence rates in athletes. This is also of importance when interpreting the results of our pooled data.

Compared with prevalence rates in the general population [3, 5], the studies in this review reported similar, higher, or lower rates for athletes depending on the discipline and investigation. All identified studies focusing on rowing, for example, found higher prevalence rates for athletes in this discipline. These studies suggested that factors such as high training volume and repetitive motions (e.g. forward flexion of the trunk depending on the stroke phase of rowing) might be responsible for the high prevalence rates. In this context, Howell [57] reported a high correlation between excessive lumbar flexion and the incidence of low back pain or discomfort in a group of elite lightweight female rowers, and suggested that mechanical stress on non-contractile tissue sufficient to stimulate pain receptors of the musculoskeletal system of the low back could result from intermittent or continuous hyperflexion of the lumbar spine. Dalichau and Scheele [58] discussed the sports-related physical requirement profile responsible for back pain. High physical loads, repetitive mechanical strain, and static or dynamic extreme body positions increase the risk of spinal overload and overuse [59].

In some sports disciplines, contact with an opponent and the resulting strain on the spine might be an additional risk factor for back pain. Back pain is often associated with sport-specific mechanical loads and injury, especially with regard to contact and combative sports [58, 60]. However, there was wide heterogeneity of prevalence data for soccer, handball, ice and field hockey, basketball, and rugby in the present review. The range of lifetime prevalence of low back pain in these disciplines was 1–64%. There was also wide variation, 3–63%, in lifetime-prevalence data for the court sports tennis and volleyball.

The time of examination must also be considered when comparing prevalence rates of back pain. Some studies collected data during the peak season while others did so during the off-season. Newlands et al. [27] showed that prevalence rates vary during an athlete’s season. They found a high variability (6–25%) of monthly low back pain prevalence during a 12-month period among international-level rowers. The highest rates were collected during the peak season. Given that prevalence rates of back pain vary during the season, the time of assessment needs to be considered when comparing the results of different studies.

### Risk Factors

Different risk factors for back pain were discussed and calculated for studies included in this systematic review. The most thoroughly investigated potential risk factors were the spinal load, age, sex, anthropometrics, and a previous history of back pain. An overview of all calculated risk factors is shown in Table 5.

**Table 5:** Calculated risk factors of high-quality studies included in the review

References	Sport discipline	Method	Risk factor	Results
Bahr et al. [29]	Cross-country skiing Rowing Orienteering Controls	Logistic regression	TV	NS
		Logistic backward stepwise regression analysis of low back pain	Age, sex, height, weight, yearly training load	Age (*p* = 0.009) and sex (*p* = 0.037) were the only parameters that influenced the results
		Logistic backward stepwise regression analysis of missed training because of low back pain	Height, weight and age, sex, yearly training load	Height (*p* = 0.027), weight (*p* = 0.072) and age (*p* = 0.068) influenced the results
Baranto et al. [49]	Weight lifting Wrestling Orienteering Ice hockey Controls	Correlation	MRI changes	NS
Brynhildsen et al. [34]	Basketball Volleyball Soccer Controls	Chi-square analysis	Oral contraceptive use	NS
Çali et al. [52]	Soccer	Chi-square analysis		NS
			Age, height, weight, BMI	
			Number of matches	NS
			Number of matches played in starting position	S (*p* < 0.05)
			Flexibility of LB muscles	NS
			Flexibility of hamstring muscles	NS
			Hamstring shortness	S (*p* < 0.05, for both sides)
			Playing position	NS
			Total active years	NS
Eriksson et al. [43]	Cross-country skiing	ANOVA, Chi-square, Fisher’s exact test	Sex Age Height Weight Off-season training Pre-season training Years raced Stretching per week Muscle strength training hours	NS S (*p* < 0.01 for women) NS NS NS NS S (*p* < 0.05 for women) S (*p* < 0.05) NS
Iwamoto et al. [39]	Rugby	Logistic regression analysis	*Radiographic abnormalities*	
			Spondylolysis	S (OR = 3.03; *p* < 0.001; 95% CI 2.58–3.57)
			Disc space narrowing	NS
			Spinal instability	NS
			Schmorl’s node	NS
			Balloon disc	NS
			Spina bifida occulta	NS
Maselli et al. [28]	Rowing	Univariate logistic regression model Mulitivariate logistic regression model with stepwise selection procedure	Sex	S (OR = 2.62; *p* = 0.03; 95% CI 1.09–6.27)
			Age (1-year increment)	NS
			BMI	NS
			Years of experience (1-year increment)	NS
			Weekly hours (1-hour increment)	NS
			Musculoskeletal disorders (yes vs. no)	NS
			Other sports	NS
			*Typology of rowing*	
			Only sculling (RG)	
			Sculling/sweep	S (OR = 4.43; *p* < 0.001; 95% CI 1.87–10.48)
			Sweep	S (OR = 3.32; *p* = 0.03; 95% CI 1.16–9.55)
			Change of typology of row (yes vs. no)	NS
			*Category*	
			Junior (RG)	
			Cadet	NS
			Youngsters	NS
			Master	NS
			Senior	NS
Newlands et al. [27]	Rowing	Multivariate logistic regression model Pearson correlations	*Relationship between potential* *RF and low back pain*	
			Age	S (OR = 1.08; *p* = 0.02; 95% CI 1.01–1.15)
			Sex	NS
			BMI	NS
			Rowing discipline	NS
			History of previous low back pain	S (OR = 2.06; *p* = 0.01; 95% CI 1.22–3.48)
			MCS score	NS
			*Relationship between training load and low back pain*	
			Total TH/mo	S (*r* = 0.83, *p* < 0.01)
			Number of ergometer TH/mo	S (*r* = 0.80, *p* < 0.01)
			Average TH/mo	S (*r* = 0.73, *p* < 0.01)
			Average number of km rowed per mo	S (*r* = 0.71, *p* = 0.01)
Ng et al. [45]	Rowing	Chi-square statistics	Sex	S (*p* < 0.001)
Swärd et al. [48]	Gymnastics	Correlation by Pitman’s test, which coincides with Fisher’s exact test	MRI findings	NS
Swärd et al. [46]	Wrestling Soccer Gymnastics Tennis	Correlation by Pitman’s test, which coincides with Fisher’s exact test	Age	S (*p* < 0.05)
			*Radiological changes*	
			Spondylolysis	NS
			Disc height reduction	S (*p* < 0.05)
			Schmorl’s nodes	S (*p* < 0.05)
			Change of configuration of vertebral body	S (*p* < 0.05)
Tunas et al. [31]	Soccer Handball	Binary logistic regression	TV	NS
			Seasons (years)	NS
			*Soccer*	
			Striker (RG)	
			Defender	NS
			Midfielder	S (*p* = 0.03; OR = 2.5; 95% CI 1.1–5.7)
			Forward	NS
			Goalkeeper	S (*p* = 0.05; OR = 3.0; 95% CI 1.0–9.3)
			*Handball*	
			Line player (RG)	
			Back	NS
			Wing	NS
			Goalkeeper	NS
van Hilst et al. [26]	Field hockey Soccer Speed skaters	Chi-square statistics Bivariate logistic regression analysis	Sex	S in field hockey: *p* = 0.01
			Age	S in speed skating: OR = 1.4; *p* < 0.05; 95% CI 1.1–1.9
			BMI	NS
			Satisfied with performance	S in field hockey: OR = 0.5; *p* < 0.05; 95% CI 0.3–0.9
			Satisfied with coaching staff	S in field hockey: OR = 0.5; *p* < 0.05; 95% CI 0.4–0.8
			Number of training hours	S in speed skating: OR = 1.1; *p* < 0.05; 95% CI 1.03–1.2
			Stretching	NS
			Core stability training	NS
			Strength training machine	NS
			Strength training weights	NS
			Performing Pilates	S in speed skating: OR = 4.1; *p* < 0.05; 95% CI 1.1–15.7
			Years of experience	NS
			Duration of warming up	S in speed skating: OR = 1.1; *p* < 0.05; 95% CI 1.02–1.1
			Having a job	NS

*ANOVA* analysis of variance, *BMI* body mass index, *CI* confidence interval, *km* kilometers, *LB* low back, *MRI* magnetic resonance imaging, *MCS* Movement Competency Screen, *mo* month, *NS* not significant, *OR* odds ratio, *RF* risk factor, *RG* reference group, *S* significant, *TH* training hours, *TV* training volume, *vs.* versus

^a^Except where otherwise indicated

#### Spinal Load

Spinal load was investigated in the studies included in this systematic review, especially with respect to training volume or experience [26–28, 43, 52], loads exerted by different types of training – such as strength training [26, 43], and sport-specific techniques or typologies [28, 39, 43]. As shown in Table 5, the results are not consistent. For example, training volume was found to be a risk factor for back pain in speed skaters [26] and rowers [27] but not in field hockey or soccer players [26] or in another investigation on rowers [28]. These different results regarding risk factors indicate that sport-specific differences might lead to different loads on the spine. Van Hilst et al. [26] found training volume to be a risk factor for speed skaters, but not for soccer and field hockey players. However, they emphasized that the examined speed skaters had a much higher weekly training volume, making comparison difficult. Additionally, the content of training and thus the sport-specific load differs among the above-mentioned sports. Sport-specific differences also become obvious when considering degenerative changes of the spine that might result from sport-specific loads. In the treatment and exercise management of back pain, the focus is often on the muscle system. However, as Belavý et al. [61] summarized in a narrative review regarding whether exercise can positively influence the intervertebral discs, the discs are also well-recognized sources of pain. A number of studies summarized by Belavý et al. [61] examined intervertebral disc degeneration and/or spinal abnormalities in specific athletic populations, and thoracic and lumbar intervertebral disc or spinal damage is more common in several different types of sports. As Belavý et al. [61] reported, this is seen in sports in which traumatic spinal injury is more frequent (e.g., gymnastics, wrestling), in sports involving repetitive loading of the spine during motion or load extremes (e.g., gymnastics, cricket, weightlifting, rowing), and in sports in which the spine is subject to high-impact loads with sometimes unpredictable landing forces (e.g., horseback riding, volleyball). Due to the nature of these sports, it is not surprising that the incidence of intervertebral disc or spine abnormalities is higher. There is also some evidence that upright activities such as running may have a protective effect on the intervertebral discs or, at an elite level, are at least less detrimental to the intervertebral discs than are other sports at an elite level. Some studies in the present review investigated the relationship between radiographic abnormalities and back pain [39, 46, 48, 49]. Although degenerative changes in the spine are not always accompanied by pain, the prevalence of back pain is increasingly affected, even if just a few athletes with sports-related spinal changes experience back pain.

#### Sex and Age

Sex and age are frequently discussed confounders regarding back pain. Most studies in the literature have reported higher prevalences for female than male athletes [17, 62–67]. This is often justified by the earlier maturity of girls or the differences in their hormonal changes during puberty compared with boys [64]. Additionally, the anatomic characteristics of the female body can reinforce the development of back pain and therefore lead to higher prevalence rates in females than in males [64]. In this context, several studies have discussed the lower muscle mass and bone densities of females that might result in destabilization of the body and thus insufficient compensation for high loads, menstrual low back pain, and pregnancy-related back pain [64, 68, 69]. However, the result of the present review did not confirm this hypothesis. Most of the studies in this review that differentiated between the two sexes found a higher prevalence of back pain in male athletes. Only Ng et al. [45] found that male rowers performed significantly more hours of training than females and that a higher training volume was linked to low back pain or injuries in rowers. Swärd et al. [46] also found higher prevalence of back pain in male athletes; however, it must be considered that the male athletes were older than the female athletes (median age of 19 vs. 16 years, respectively), and this could be an age-related effect instead of a sex-related effect.

Differences in the prevalence of musculoskeletal pain between the sexes might also be influenced by different factors. In some disciplines, male athletes might tolerate higher loads because of their higher training volume, higher loads during strength training, or differences in basic rules (e.g., the number of sets in tennis). Additionally, differences in spinal kinematics have been reported for some disciplines, and spinal kinematics are suggested to be associated with back pain.

Another frequently discussed confounder for back pain is age. In the general population, the prevalence of back pain in children and adolescents is reportedly lower than that in adults, but is rising [18, 70]. Although calculation of age-related effects on back pain was not possible for all studies in the present review, some of the investigated studies calculated the effects of age on its own. Cali et al. [52], Maselli et al. [28], and Ng et al. [45] did not find age to be a risk factor for back pain, while other researchers did [26, 27, 29, 43]. Some studies discussed the high vulnerability of the spine in growing individuals as a risk factor for back pain in young athletes and were therefore interested in the prevalence of back pain in adolescent athletes. However, these studies did not compare their results with an older population.

#### Anthropometrics

Various anthropometric parameters were examined as risk factors for back pain, including height, weight, and body mass index, and the results varied among the studies. At a high competition level, anthropometrics usually differ among different disciplines. For example, basketball players or rowers usually are taller than gymnasts. On the one hand, athletes with a stature that is typical in their discipline (e.g., tall volleyball players) better meet the requirements to become successful (compared to small volleyball players). On the other hand, participation in different sports generally leads to adaption by differently stressed body structures. For example, the bodies of weight lifters greatly differ from those of gymnasts. Accordingly, it is difficult to interpret anthropometric parameters as risk factors in some disciplines. We are therefore unable to determine whether an anthropometric parameter or a sport-specific load is responsible for a back pain problem.

### Study Limitations

This review did not cover all studies concerning back pain prevalence in sports, it was limited to articles written in English or German and published in scientific journals. Other sources of information such as abstracts and reports were not considered. It was also not a blinded review because the different studies were easily recognizable. However, the reviewers had no personal or professional ties to any of the authors of the articles reviewed. The two reviewers gave independent assessments of the articles and disagreements were discussed until consensus was reached. Each article was judged solely on the basis of the full text; the original authors were not contacted for further details. The methodological measurement instrument was taken from a prevalence study by Lebouef-Yde and Lauritsen [2], who devised this instrument and arbitrarily set the threshold for acceptability at 75%. Based on different instruments, prevalence reviews were assessed and demonstrated varying thresholds for acceptability. Some studies set the threshold for acceptability absolutely arbitrarily, others set no cutoff-point, and several studies used the mean score for all studies in the review as threshold for acceptability. Louw et al. [4] used the same tool as that used in the present review. As in their review, we used a cutoff-point based on a mean methodological quality assessment score, which might have influenced the results. There were some differences between the results of high- and low-quality studies. For example, the overall lifetime prevalence of low back pain was higher in low-quality than in high-quality studies. Fewer low-quality studies demonstrated low prevalence rates, so that the range was 53–92%. Quality assessment of studies that are included in systematic reviews is important but is also considered a source of scientific controversy. Quality scores can be misleading because there is no objective way to assess quality, and different methods lead to different analytic results. It is also difficult to determine how to weight each item in an overall quality score. However, it has been suggested that sum scores in a systematic review can help to distinguish between studies with low versus high risk of bias [11]. Nevertheless, the tool we used in this review must be applied critically. Although it focuses exactly on the topic of our interest, it addresses the epidemiology of back pain, which involves the general population and not a special population like athletes. In particular, the items focusing on the representativeness of the sample scored conspicuously poorly. Further research should develop a tool that is more precise for athletes. For example, it should assess the definition of athletes and differentiate more precisely the details of training and competition.

The data pooling in this review must also be interpreted with caution. A comparison between representative samples that considers the exact distribution of disciplines among athletes is needed to formulate a general statement of back pain in athletes and in the general population. However, this review gives a first indication that there is in an increased risk of back pain in athletes of some disciplines.

## Conclusion

In the current review, we examined the literature on prevalence and risk factors for back pain in Olympic sports. The methodological heterogeneity of the included studies showed a wide range of prevalence rates and did not enable a detailed comparison of data among different sports, within one discipline, or versus the general population. Based on the results of this review, however, it seems obvious that back pain requires further study in some sports. Back pain seems to be a problem in some sports that have been thoroughly investigated, while other sports have been less frequently investigated or even not investigated at all. These sports might have a preventive effect on the development of back pain, which also requires clarification in further research. This would offer the opportunity for high-risk sports to positively influence back pain or even prevent it. Our comparison with the general population provides the first data indicating that some sports seem to have a higher risk for back pain. However, as many studies in the literature suggest, a sedentary lifestyle also leads to higher prevalence rates. The optimal dose-effect relationship in sports remains unclear and needs to be examined in further research. Also the influence of uninvestigated factors, such as psychosocial factors, needs further examination. This review is the basis for future development of sport-specific back pain prevention strategies. For this purpose, it is additionally important to understand exactly what type of strain in which sports involves the spine and whether this strain is detrimental or beneficial for the spine. In general, when comparing the prevalence of back pain in different sports, it is also of importance to consider sport-specific characteristics that might influence prevalence rates. These characteristics are due to differences in the contents of training and competition, body anthropometrics, and the age of peak competitive performance. Further research should more precisely focus on the differences in sports disciplines and their specific risk factors using identical tools for data collection. This would provide the opportunity to develop special prevention strategies for back pain. Additionally, athletes, coaches, physicians, and physiotherapists should be sensitized to the back pain problem in athletes and seek to integrate prevention programs in daily training.

## Electronic supplementary material

Below is the link to the electronic supplementary material.

Supplementary material 1 (PDF 38 kb)

Supplementary material 2 (PDF 89 kb)

Supplementary material 3 (PDF 54 kb)
