# Supplementary material for: Prevalence of Back Pain in Sports: A Systematic Review of the Literature
Source: Sports Med. 2016 Dec 29;47(6):1183–207. doi: 10.1007/s40279-016-0645-3 (PMC5432558; doi:10.1007/s40279-016-0645-3)
Supplement: Supplementary file 1 — Supplementary material 1 (PDF 38 kb) [file 40279_2016_645_MOESM1_ESM.pdf]

Electronic Supplementary Material Table S1 Search strategy for all databases

#1	back pain
#2	spine
#3	neck pain
#4	Search (#1) and sports and prevalence
#5	Search (#1) and alpine and skiing
#6	Search (#1) and aquatics
#7	Search (#1) and archery
#8	Search (#1) and badminton
#9	Search (1#) and basketball
#10	Search (1#) and boxing
#11	Search (1#) and biathlon
#12	Search (1#) and bobsleighing
#13	Search (1#) and canoe
#14	Search (1#) and cross-country and skiing
#15	Search (1#) and curling
#16	Search (1#) and cycling
#17	Search (1#) and equestrian
#18	Search (1#) and fencing
#19	Search (1#) and figure and skating
#20	Search (1#) and football
#21	Search (1#) and freestyle and skiing
#22	Search (1#) and golf
#23	Search (1#) and gymnastics
#24	Search (1#) and handball
#25	Search (1#) and hockey
#26	Search (1#) and horse and riding
#27	Search (1#) and ice and hockey
#28	Search (1#) and judo
#29	Search (1#) and luge
#30	Search (1#) and nordic and combined
#31	Search (1#) and pentathlon
#32	Search (1#) and rugby
#33	Search (1#) and running
#34	Search (1#) and sailing
#35	Search (1#) and shooting
#36	Search (1#) and short and track
#37	Search (1#) and ski and jumping
#38	Search (1#) and snowboarding
#39	Search (1#) and soccer
#40	Search (1#) and speed and skating
#41	Search (1#) and swimming
#42	Search (1#) and table and tennis
#43	Search (1#) and taekwondo
#44	Search (1#) and tennis
#45	Search (1#) and track and field
#46	Search (1#) and trampoline
#47	Search (1#) and triathlon
#76	Search (2#) and judo
#77	Search (2#) and luge
#78	Search (2#) and nordic and combined
#79	Search (2#) and pentathlon
#80	Search (2#) and rugby
#81	Search (2#) and running
#82	Search (2#) and sailing
#83	Search (2#) and shooting
#84	Search (2#) and short and track
#85	Search (2#) and ski and jumping
#86	Search (2#) and snowboarding
#87	Search (2#) and soccer
#88	Search (2#) and speed and skating
#89	Search (2#) and swimming
#90	Search (2#) and table and tennis
#91	Search (2#) and taekwondo
#92	Search (2#) and tennis
#93	Search (2#) and track and field
#94	Search (2#) and trampoline
#95	Search (2#) and triathlon
#96	Search (2#) and volleyball
#97	Search (2#) and water and polo
#98	Search (2#) and wrestling
#99	Search (3#) and sports and prevalence
#100	Search (3#) and alpine and skiing
#101	Search (3#) and aquatics
#102	Search (3#) and archery
#103	Search (3#) and badminton
#104	Search (3#) and basketball
#105	Search (3#) and boxing
#106	Search (3#) and biathlon
#107	Search (3#) and bobsleighing
#108	Search (3#) and canoe
#109	Search (3#) and cross-country and skiing
#110	Search (3#) and curling
#111	Search (3#) and cycling
#112	Search (3#) and equestrian
#113	Search (3#) and fencing
#114	Search (3#) and figure and skating
#115	Search (3#) and football
#116	Search (3#) and freestyle and skiing
#117	Search (3#) and golf
#118	Search (3#) and gymnastics
#119	Search (3#) and handball
#120	Search (3#) and hockey
#121	Search (3#) and horse and riding
#122	Search (3#) and ice and hockey

Electronic Supplementary Material Table S1 continued

#48	Search (1#) and volleyball	#123	Search (3#) and judo
#49	Search (1#) and water and polo	#124	Search (3#) and luge
#50	Search (1#) and wrestling	#125	Search (3#) and nordic and combined
#51	Search (1#) and weightlifting	#126	Search (3#) and pentathlon
#52	Search (2#) and sports and prevalence	#127	Search (3#) and rugby
#53	Search (2#) and alpine and skiing	#128	Search (3#) and running
#54	Search (2#) and aquatics	#129	Search (3#) and sailing
#55	Search (2#) and archery	#130	Search (3#) and shooting
#56	Search (2#) and badminton	#131	Search (3#) and short and track
#57	Search (2#) and basketball	#132	Search (3#) and ski and jumping
#58	Search (2#) and boxing	#133	Search (3#) and snowboarding
#59	Search (2#) and biathlon	#134	Search (3#) and soccer
#60	Search (2#) and bobsleighing	#135	Search (3#) and speed and skating
#61	Search (2#) and canoe	#136	Search (3#) and swimming
#62	Search (2#) and cross-country and skiing	#137	Search (3#) and table and tennis
#63	Search (2#) and curling	#138	Search (3#) and taekwondo
#64	Search (2#) and cycling	#139	Search (3#) and tennis
#65	Search (2#) and equestrian	#140	Search (3#) and track and field
#66	Search (2#) and fencing	#141	Search (3#) and trampoline
#67	Search (2#) and figure and skating	#142	Search (3#) and triathlon
#68	Search (2#) and football	#143	Search (3#) and volleyball
#69	Search (2#) and freestyle and skiing	#144	Search (3#) and water and polo
#70	Search (2#) and golf	#145	Search (3#) and wrestling
#71	Search (2#) and gymnastics	#146	Search (3#) and weightlifting
#72	Search (2#) and handball		
#73	Search (2#) and hockey		
#74	Search (2#) and horse and riding		
#75	Search (2#) and ice and hockey		
