# Supplementary material for: Prevalence of Back Pain in Sports: A Systematic Review of the Literature
Source: Sports Med. 2016 Dec 29;47(6):1183–207. doi: 10.1007/s40279-016-0645-3 (PMC5432558; doi:10.1007/s40279-016-0645-3)
Supplement: Supplementary file 2 — Supplementary material 2 (PDF 89 kb) [file 40279_2016_645_MOESM2_ESM.pdf]

Electronic Supplementary Material Table S2 Characteristics of low-quality studies included in the review

Reference	Country	Final sample size	Age [years] mean ± SD[a] (range)	Level	Response rate [%]	Collection-mode	Definition of pain	Localization	Recall periods	Prevalence [%]
**Basketball**										
Greene et al. 2001 [73]	USA	19 (M)<br>14 (F)	19 ± 1	Varsity athletes	-	Q	Low back injury in the last 5 years, an injury was defined as any LBP that caused an athlete to miss or not participate fully in at least three practice sessions or competitions and that resulted in a visit to a sports physician (definition Greene et al.)	LB	5-yr, 1-yr	5-yr: 32, 1-yr: 32<br>5-yr: 21, 1-yr: 0
Hangai et al. 2009 [40]	Japan	63 (M: 44, F: 19)	20 ± 1	Well-trained university athletes, > 5 yrs experience in their sport	-	Q	-	LB	LT, 4-wk	LT: 81, 4-wk: 18
**Canoeing**										
Willscheid et al. 2014 [81]	Germany	63 (M)<br>41 (F)	19<br>18	> 10 h/wk	57	Q	-	LB	last 4 yr	33
**Cross-country**										
Greene et al. 2001 [73]	USA	15 (M)<br>24 (F)	19 ± 1	Varsity athletes	-	Q	See definition Greene et al.	LB	5-yr, 1-yr	5-yrs: 0, 1-yr: 7<br>5-yrs: 8, 1-yr: 0
**Diving**										
Greene et al. 2001 [73]	USA	3 (F)	19 ± 1	Varsity athletes	-	Q	See definition Greene et al.	LB	5-yr, 1-yr	5-yr: 100, 1-yr: 0
**Fencing**										
Greene et al. 2001 [73]	USA	14 (M)<br>17 (F)	19 ± 1	Varsity athletes	-	Q	See definition Greene et al.	LB	5-yr, 1-yr	5-yrs: 0, 1-yr: 0<br>5-yrs: 18, 1-yr: 6
**Field hockey**										
Greene et al. 2001 [73]	USA	23 (F)	19 ± 1	Varsity athletes	-	Q	See definition Greene et al.	LB	5-yr, 1-yr	5-yrs: 13, 1-yr: 0
Lindgren and Twomey 1988 [75]	Australia	32 (M: 15, F: 17)	17-26	Elite, all were members of the 1985 Australian Institute of Sport hockey squad	-	Q	-	LB	LT	78
Murtaugh 2001 [35]	Canada	158 (F)	20 (14–32)	High school (30), intermediate level (23), varsity (75), national level (30)	100	Q	-	B	LT	59
Reilly and Seaton 1990 [77]	England	81	-	Local hockey clubs	81	Q	-	LB	LT	53

Electronic Supplementary Material Table S2 continued

Reference	Country	Final sample size	Age [years] mean ± SD[a] (range)	Level	Res-ponse rate [%]	Colle-ction-mode	Definition of pain	Loca-liza-tion	Recall periods	Prevalence [%]
**Golf**										
Greene et al. 2001 [73]	USA	22 (M)<br>9 (F)	19 ± 1	Varsity athletes	-	Q	See definition Greene et al.	LB	5-yr, 1-yr	5-yrs: 18, 1-yr: 0<br>5-yrs: 22, 1-yr: 11
Vad et al. 2004 [80]	USA	42 (M)	31 (21-40)	Elite	-	E	LBP without lower extremity radicular symptoms limiting their golf performance for greater than 2 wks within the past yr	LB	past yr	33
**Gymnastics**										
Greene et al. 2001 [73]	USA	11 (F)	19 ± 1	Varsity athletes	-	Q	See definition Greene et al.	LB	5-yr, 1-yr	5-yrs: 36, 1-yr: 9
Koyama et al. 2013 [36]	Japan	104 (M: 70, F: 34)	20 ± 1	17 pre-elite, 71 elite, 16 national, TV: 4h for 6d/wk	-	Q	OCU-Test with a total score of 1 point or more was considered to have LBP	LB	PP	49
Mulhearn and George 1999 [76]	Canada	T: 22<br>12 (M)<br>10 (F)	 17 ± 2<br>13 ± 1	Competing nationally or internationally for at least 3 yrs, TV: M: 6 + 1d/wk; F: 5 + 1 d/wk	-	Q	-	LB	5-yr	50<br>75<br>20
Szot et al.1985 [78]	Poland	41 (M)	21 (15-31)	Members of the national gymnastics team	-	Q	-	LB	-	49
**Horse riding**										
Kernahan et al. 1979 [74]	UK	38	15-30	-	-	Q	-	LB	LT	92
**Ice hockey**										
Greene et al. 2001 [73]	USA	29 (M)<br>18 (F)	19 ± 1	Varsity athletes	-	Q	See definition Greene et al.	LB	5-yr, 1-yr	5-yrs:14, 1-yr: 17<br>5-yrs:11, 1-yr: 11
**Judo**										
Okada et al. 2007 [37]	Japan	T: 82 (M)<br>Lightweight: 29<br>Middleweight: 31<br>Heavyweight: 22	20 ± 1<br>20 ± 1<br>20 ± 1<br>21 ± 1	Elite collegiate judo athletes, TV: 6d/wk for approximately 3h/d	-	Q	Subjects with a total of 1 or more points on the OCU Test was considered to have nonspecific LBP	LB	PP	35<br>35<br>32<br>41
**Rowing**										
Howell 1984 [57]	USA	17 (F)	-	Elite, participants of the National Development Camp of the US Rowing Association	-	Q	Occasional or chronic low backache or discomfort	LB	LT I	82
**Running**										
Hangai et al. 2009 [40]	Japan	43 (M: 33, F: 10)	19 ± 1	Well-trained university athletes, > 5 yrs experience in their sport	-	Q	-	LB	LT, 4-wk	LT: 91, 4-wk: 37

Electronic Supplementary Material Table S2 continued

Reference	Country	Final sample size	Age [years] mean ± SD[a] (range)	Level	Res-ponse rate [%]	Colle-ction-mode	Definition of pain	Loca-liza-tion	Recall periods	Prevalence [%]
**Soccer**										
Brynhildsen et al. 1997 [72]	Sweden	261 (F)	21 (15-28)	First to third division in Östergötland, Sweden	-	Q	Experience of BP during the last active soccer playing season but did not have to prevent the woman from her daily activities or from taking part in practice sessions or games	LB	last season	29
Greene et al. 2001 [73]	USA	29 (M)<br>21 (F)	19 ± 1	Varsity athletes	-	Q	See definition Greene et al.	LB	5-yr, 1-yr	5-yrs: 17, 1-yr: 10<br>5-yrs: 5, 1-yr: 5
Hangai et al. 2009 [40]	Japan	47 (M)	19 ± 0	Well-trained university athletes, > 5 yrs experience in their sport	-	Q	-	LB	LT, 4-wk	LT: 77, 4-wk: 25
**Swimming**										
Greene et al. 2001 [73]	USA	35 (M)<br>23 (F)	19 ± 1	Varsity athletes	-	Q	See definition Greene et al.	LB	5-yr, 1-yr	5-yrs: 3, 1-yr: 0<br>5-yrs: 13, 1-yr: 13
Hangai et al. 2009 [40]	Japan	47 (M: 38, F: 9)	20 ± 1	Well-trained university athletes, > 5 yrs experience in their sport	-	Q	-	LB	LT, 4-wk	LT: 76, 4-wk: 36
Kaneoka et al. 2007 [38]	Japan	56 (M: 35, F: 21)	20 (15-27)	Elite swimmers, participants of the national training camps, TV: 49047 meter/wk	-	Q	Severe LBP: that interfered with their daily living activities or hampered their training	LB	LT	77, serve LBP: 52
		38 (M: 24, F: 14)	21 (18-24)	students who belonged to a recreational swim club	-	Q				87, serve LBP: 37
Martins et al. 2014 [41]	Brazil	T: 42<br>52% (M)<br>48% (F)	23 ± 4	Participated at world championships, TV: 46 ± 19 km/wk swimming	-	Q	-	LB	PP	10<br>0<br>20
**Tennis**										
Greene et al. 2001 [73]	USA	16 (M)<br>10 (F)	19 ± 1	Varsity athletes	-	Q	See definition Greene et al.	LB	5-yr, 1-yr	5-yrs: 13, 1-yr: 0<br>5-yrs: 40, 1-yr: 20
Vad et al. 2003 [79]	USA	100 (M)	25 (17-37)	Professionals	-	E	LBP limiting tennis performance for greater than 2 weeks	LB	-	40
**Track and field**										
Aggrawal et al. 1979 [25]	India	25 (M)	27 (17-32)	Activity for more than 6 months	-	E	-	B	-	48
Greene et al. 2001 [73]	USA	39 (M)<br>23 (F)	19 ± 1	Varsity athletes	-	Q	See definition Greene et al.	LB	5-yr, 1-yr	5-yrs: 15, 1-yr: 8<br>5-yrs: 13, 1-yr: 0

Electronic Supplementary Material Table S2 continued

Reference	Country	Final sample size	Age [years] mean ± SD[a] (range)	Level	Response rate [%]	Collection-mode	Definition of pain	Localization	Recall periods	Prevalence [%]
**Volleyball**										
Greene et al. 2001 [73]	USA	27 (F)	19 ± 1	Varsity athletes	-	Q	See definition Greene et al.	LB	5-yr, 1-yr	5-yrs: 22, 1-yr: 7
**Weightlifting**										
Aggrawal et al. 1979 [25]	India	25 (M)	32 (17-38)	Activity for more than 6 months	-	E	-	B	-	40

B=back; BP=back pain; d=day; E=examination; F=female; h=hour; I=Incidence; km=kilometer; LB=low back; LBP=low back pain; LT=lifetime; M=male; OCU= Osaka City University; PP=point prevalence; Q=questionnaire; T=total; TV=training volume; USA=United States of America; UK=United Kingdom; US=United States; wk=week; yr=year; a Except where otherwise indicated
