# Supplementary material for: Prevalence of Back Pain in Sports: A Systematic Review of the Literature
Source: Sports Med. 2016 Dec 29;47(6):1183–207. doi: 10.1007/s40279-016-0645-3 (PMC5432558; doi:10.1007/s40279-016-0645-3)
Supplement: Supplementary file 3 — Supplementary material 3 (PDF 54 kb) [file 40279_2016_645_MOESM3_ESM.pdf]

Electronic Supplementary Material Table S3 Included high-quality studies comparing athletes with controls

Reference	Sports discipline	Final sample size	Age [years] mean ± SD[a] (range)	Level	Res-pon-se [%]	Loca-liza-tion	Recall periods	Prevalence [%]	Comments
Alricsson and Werner 2005 [50]	Cross-country skiing	120 (M: 58%, F: 42%)	18 ± 1	Top national level	92	B, LB, TH, C	LT, 3-mo	LT: B: 47, LB: 44, TH: 7, C: 3;<br>3-mo: B: 26, C: 11	- Significantly higher 3-mo prevalence for BP (p = 0.02) and NP (p = 0.01) in controls compared to athletes
	**Controls**	**993 (M: 49%, F: 51%)**	**18 ± 1**	**High school students**	**68**	**B, C**	**3-mo**	**3-mo: B: 37, C: 21**	
Bahr et al. 2004 [29]	Cross-country skiing	257 (M: 165, F: 92)	M: 23 ± 5, F: 21 ± 4	Qualified for national championships	100	LB	LT, 12-mo, 7-d	LT: 65, 12-mo: 63, 7-d: 24	- Significantly higher LT prevalence rates in skiers and rowers than in controls,
	Rowing	199 (M: 131, F: 68)	M: 21 ± 6, F: 22 ± 5		100			LT: 63, 12-mo: 55, 7-d: 25	- Significantly higher 12-month prevalence for skiers than for controls
	Orienteering	227 (M: 129, F: 98)	M: 24 ± 7, F: 23 ± 6		99			LT: 57, 12-mo: 50, 7-d: 19	
	**Controls**	**158 (M: 95, F: 63)**	**M: 24 ± 5, F: 22 ± 4**		**66**			**LT: 51, 12-mo: 47, 7-d: 20**	
Baranto et al. 2009 [49]		T: 71 (M)	26 (18-41)	Swedish top male athletes, active at least since the age of 10 years	-	B	LT	78	- Obvious difference between athletes and controls, with LT prevalence of 78 % and 38 %, respectively
	Weight lifting	21	Med: 30 (18-40)					71	
	Wrestling	13	Med: 24 (22-41)					77	
	Orienteering	18	Med: 25 (20-35)					56	- They did not mention if these results were significant
	Ice hockey	19	Med: 24 (19-31)					90	
	**Controls**	**21**	**Med: 28 (22-38)**	**Non-athletes, randomly selected**				**38**	
Brynhildsen et al. 1997 [34]	Basketball	150 (F)	Med: 21 (16-31)	Swedish first league clubs	85	LB	LT, 7-d	LT: 53, 7-d: 21	- Significantly higher LT prevalence in volleyball and basketball players compared to soccer players and controls (p< 0.05)
	Volleyball	205 (F)	Med: 22 (16-35)					LT: 63, 7-d: 34	
	Soccer	361 (F)	Med: 21 (14-36)					LT: 42, 7-d: 32	
	**Controls**	**113 (F)**	**Med:20 (15-36)**		75			**LT: 43, 7-d: 18**	- Significantly higher PP among athletes than controls (p< 0.01)
Haydt et al. 2012 [47]	Field hockey	90 (F)	19 ± 1 (18-22)	NCAA Division III intercollegiate	-	LB	I	56	- No significant differences between athletes and controls
	**Controls**	**98 (F)**	**20 ± 1 (18-24)**	**age-matched control group**				**55**	
Selanne et al. 2014 [54]	Ice hockey	121 (M)	15 (14-16)	National level	93	LB, UB, N	3-mo	LB: 54, UB: 31, N: 44	- Significantly higher LBP prevalence in athletes than controls
	**Controls**	**618 (M)**		**Age-matched school boys**				**LB: 35, UB: 29, N: 48**	
Swärd et al. 1991 [48]	Gymnastics	24 (M)	Med: 23 (19-29)	Present or previous members of the Swedish national team	-	B	LT , PP	LT: 79, PP: 38	- Significantly higher BP LT prevalence in gymnastics than controls (p< 0.001)
	**Controls**	**16 (M)**	Med: 26 (23-36)	**Non-athletes, randomly selected**				**LT: 38, PP: 0**	
Tunas et al. 2014 [31]	Football	277 (F)	22 ± 4 (18-32)	Elite	98	LB	LT, 12-mo, 7-d	LT: 61, 12-mo: 57, 7-d: 24	- No significant differences between athletes and controls
	Handball	190 (F)	22 ± 3 (18-32)		99			LT: 63, 12-mo: 59, 7-d: 26	
	**Control**	**167 (F)**	**26 ± 4 (18-32)**	**Randomly selected**	**43**			**LT: 65, 12-mo: 60, 7-d: 31**	

B=back; BP=back pain; C=cervical spine; NCAA=National Collegiate Athletic Association; d=day; F=female; I=Incidence; LBP=low back pain; LT=lifetime; M=male; mo=month; Med=median; N=neck; NP=neck pain; PP=point prevalence; SD[a]=standard deviation; T=total; TH=thoracic; UB=upper back; a Except where otherwise indicated
